# Wilms’ Tumor: A Review of Clinical Characteristics, Treatment Advances, and Research Opportunities

**DOI:** 10.3390/medicina61030491

**Published:** 2025-03-12

**Authors:** Mihai Cristian Neagu, Vlad Laurenţiu David, Emil Radu Iacob, Sorin Dan Chiriac, Florin Lucian Muntean, Eugen Sorin Boia

**Affiliations:** 1 Department of Pediatric Surgery and Orthopedics, Faculty of Medicine, “Victor Babes” University of Medicine and Pharmacy Timisoara, 2nd Eftimie Murgu Square, 300041 Timisoara, Romania; mihai.neagu@umft.ro (M.C.N.); david.vlad@umft.ro (V.L.D.); radueiacob@umft.ro (E.R.I.); boia.eugen@umft.ro (E.S.B.); 2 Department X—Surgery II, Faculty of Medicine, “Victor Babes” University of Medicine and Pharmacy Timisoara, 2nd Eftimie Murgu Square, 300041 Timisoara, Romania; munteanu.florin@umft.ro

**Keywords:** nephroblastoma, Wilms’ tumor, pediatric oncology, treatment therapies

## Abstract

Nephroblastoma is a complex childhood cancer with a generally favorable prognosis, well-defined incidence, and demographic profile but with significant challenges in terms of recurrence and long-term health outcomes. Although the management of this pathology has evolved, leading to improved survival rates, continued research into the long-term effects of treatment and the genetic factors influencing its development is still required. The survival landscape for Wilms tumor is evolving, with emerging research focusing on therapeutic biomarkers and genetic predispositions that influence treatment efficacy and survival rates. Identifying predictors for treatment response, such as specific genetic markers and histologic features, emerges as a critical area of study that could refine future interventions. The management of Wilms tumor is complex, taking into account the stage of the disease, histological classification, and individual patient factors, including age and the presence of syndromic associations. As treatment paradigms evolve, the integration of precision medicine approaches may enhance the ability of clinicians to personalize treatment to improve long-term survival outcomes for a broader range of patients. Recent advances in technology, including machine-learning approaches, have facilitated the identification of therapeutic biomarkers that correlate with clinical outcomes. This innovative method enhances the ability to integrate clinical and genetic data to predict disease trajectory and therapeutic response.

## 1. Introduction

Nephroblastoma, also known as Wilms’ tumor, is the most common malignant kidney tumor in children, accounting for more than 90% of pediatric renal tumors [1,2]. This embryonal tumor usually arises from the metanephric blastema, which is the precursor tissue of the kidney and is characterized by a triphasic histological pattern comprising blastemal, stromal, and epithelial components [3,4]. Nephroblastoma predominantly affects children under the age of five, with about 90% of diagnoses occurring before the age of three [5,6]. The clinical presentation often includes a palpable abdominal mass, which is usually painless and may be accompanied by other symptoms such as hematuria or hypertension [6,7]. The classification of pediatric nephroblastomas and their counterparts—non-Wilms renal tumors (NWRT) is essential for accurate diagnosis and treatment planning. As stated above, histologically, nephroblastomas are characterized by a triphasic pattern of blastemal, epithelial, and stromal components, which may vary significantly between different tumors [8,9]. For example, clear cell renal sarcoma (CCRS) shows a distinct mesenchymal stromal pattern, differentiating it from other nephroblastomas [10]. In addition to Wilms’ tumor, other pediatric renal tumors include clear cell renal sarcoma (CCRS) and malignant rhabdoid tumor of the kidney (MRTK), which, although uncommon, have aggressive behavior and distinct clinical features. CCRS accounts for about 3% of pediatric kidney tumors and is known for its tendency to metastasize, especially to bone [11]. MRTK, although rare, is one of the most aggressive pediatric tumors, often presenting with metastatic disease at diagnosis [12,13]. Both CCRS and MRTK are essential to differentiate Wilms tumor from other tumors due to the different treatment protocols and prognostic outcomes [14]. 

The etiology of nephroblastoma is multifactorial, with genetic predisposition playing a significant role. Certain congenital syndromes are associated with an increased risk of developing nephroblastoma [15,16]. Tumor pathogenesis involves complex molecular mechanisms, including the deregulation of oncogenes and tumor suppressor genes that contribute to tumor growth and metastasis. Recent studies have highlighted the involvement of long non-coding RNAs (lncRNAs) in tumor biology, although the specific roles of these molecules are still being elucidated [17]. The presence of specific genetic markers and microRNAs, such as the down-regulation of miR-194 in nephroblastomas, suggests a unique pathogenesis that may influence therapeutic strategies [18,19].

Imaging techniques such as diffusion-weighted MRI are very important in distinguishing the different histopathologic subtypes of nephroblastoma and NWRT [20,21]. These imaging modalities can provide information about the tumor microenvironment and help predict responses to neoadjuvant chemotherapy, thereby improving risk stratification and personalized treatment approaches [21]. In addition, the identification of transcription factors such as LIM1, which is absent in multicystic renal dysplasia but reactivated in nephroblastomas, highlights the molecular complexity of these tumors and their developmental origins [22].

In general, the clinical presentation of Wilms’ tumor is characterized by a palpable abdominal mass, potential haematuria, and a variety of uncommon symptoms. The diverse manifestations of the tumor require a comprehensive approach to diagnosis and treatment, ensuring that both typical and atypical presentations are adequately addressed. Treatment of nephroblastoma usually involves a multimodality approach, including surgery, chemotherapy, and sometimes radiotherapy. The standard treatment protocol varies by region, with the International Society of Pediatric Oncology (ISPO) and the National Wilms Tumor Study (NWTS) providing guidelines that have significantly improved survival rates [16,23]. In developed countries, the overall survival rate for nephroblastoma can exceed 90%, while in developing regions, these rates are often lower due to various healthcare challenges [24]. The prognosis is influenced by several factors, including the histologic subtype of the tumor, the stage at diagnosis, and the presence of anaplastic features, which are associated with poorer outcomes [23].

The purpose of the present review is to provide a comprehensive overview of Wilms’ tumor, focusing on the general aspects of this malignancy (prevalence, incidence, epidemiology, characteristics, and classification) as well as on current and alternative treatment therapies by synthesizing the existing literature, with a focus on the latest findings.

## 2. Prevalence and Epidemiology of Nephroblastomas

Wilms tumor represents a significant entity in pediatric oncology, primarily because of its status as the most common kidney cancer in children, and is a notable component of the broader landscape of childhood tumors. It is frequently identified as the most common type of kidney cancer in pediatric patients [25]. Globally, nephroblastoma (Wilms tumor) accounts for about 5–7% of all childhood cancers and about 90% of pediatric renal tumors [2,26,27]. The incidence of Wilms’ tumor varies geographically, with estimates suggesting that about 1 in 10,000 children are affected globally and about 7 new cases per million children in the United States, with the majority of cases diagnosed in children under the age of 5, peaking around the age of 3 [26,28,29,30]. This tumor is characterized by a favorable prognosis, especially in developed countries, where the overall survival rate exceeds 90% for localized cases and about 70% for metastatic cases [23,31]. However, there are disparities in survival rates worldwide, with developing countries reporting significantly lower survival rates due to various factors, including limited access to healthcare and advanced treatment options [24,32].

Compared to other childhood cancers, Wilms’ tumor occupies a unique position due to its renal origin and the age demographic it affects. Given that most cases occur in children between the ages of 3 and 4, the median age of diagnosis was found to be around 38 months [30,33]. Epidemiologically, Wilms’ tumor is recognized as the sixth most common malignancy in children, with approximately 500 new diagnoses each year in the United States alone [34]. This prevalence puts it in competition with other pediatric cancers, such as leukemia and lymphoma, which remain the predominant cancers affecting children [35]. Specifically, the data show that cancers such as acute lymphoblastic leukemia rank higher in terms of incidence, accounting for about 30% of all childhood cancers [36]. Incidence rates of Wilms tumor suggest that it affects about 1 in 10,000 children in Western populations [37]. This incidence underlines its relevance to childhood cancers, especially given its specificity as a single organ mainly affecting the kidneys. In comparison, rates for other cancers, such as neuroblastoma and rhabdo-myosarcoma, show a different prevalence but highlight the diverse nature of pediatric malignancies [34,38]. Although Wilms tumor may not have the highest incidence rate, its distinct characteristics—being mainly renal and having developmental abnormalities—place it in a unique category among pediatric tumors.

Although secondary malignancies are a major concern after Wilms tumor treatment, survival rates for Wilms tumor survivors are generally lower compared to those of survivors of other types of pediatric cancers, such as neuroblastoma, indicating different long-term outcomes related to these diseases [38,39].

The epidemiology of nephroblastoma reveals that it is more common in certain demographic groups, with a notable prevalence among children of African descent [24]. Epidemiologic data indicate that nephroblastoma is predominantly diagnosed in children, with a slight female predominance, in line with some studies that report a sex ratio of around 3:2 in specific cohorts [40]. The case distribution of nephroblastoma shows that it is the most common genitourinary tumor in pediatric patients, with a reported incidence of approximately 87.9% among pediatric genitourinary tumors [41]. In addition, it has been observed that nephroblastoma may arise from genetic predisposition, with certain chromosomal abnormalities, such as 2q terminal deletions, being associated with a higher incidence of the disease [42]. Ethnic variations in incidence and outcomes suggest that genetic, environmental, and socioeconomic factors may influence disease manifestation and prognosis [24,43]. Research indicates that there are significant differences in Wilms tumor treatment outcomes between high-income countries and low- and middle-income countries. Survival rates for people with Wilms tumor have been observed to be over 90% in high-income countries, while for low-income countries, survival rates are often below 50% due to various challenges such as late presentations for treatment, malnutrition, and treatment dropout due to increased costs [44,45,46]. Efforts to improve treatment adherence and reduce dropout rates due to socioeconomic barriers are crucial for improving survival outcomes in low-income, underdeveloped regions [46,47]. In addition, the biological behavior of the tumor may vary, with high-risk subtypes, such as those with anaplastic histology, showing lower survival rates [23,48].

Recent advances in treatment modalities, including nephrectomy, chemotherapy, and radiotherapy, have significantly improved survival rates over the last few decades, exceeding 90% for localized cases [23,31,49]. However, treatment can lead to late effects, particularly in patients undergoing radiotherapy, where the incidence of secondary malignant tumors is reported to be as high as 61% in those treated with radiation, compared with only 9% in those treated with surgery and/or chemotherapy alone [50]. Although the prognosis for Wilms tumor is generally favorable [51], long-term outcomes and survival rates can fluctuate significantly based on several factors, including staging at diagnosis and response to treatment [52]. This establishes the need for continuous monitoring of Wilms tumor survivors for possible secondary malignancies, a risk observed in comparison with other childhood tumors, especially neuroblastoma [38]. Moreover, management strategies for Wilms tumor survivors are needed due to the risk of recurrence, which remains a critical concern, with approximately 15% of patients experiencing recurrence, which is associated with a high mortality rate [53]. In addition, the long-term effects of treatment can lead to chronic health problems, requiring ongoing supervision and supportive care for survivors [23,49]. Due to advances in surgical techniques and postoperative care, treatment outcomes have improved over the decades, encouraging an increased clinical focus on longitudinal studies that can reveal more about survival and quality of life after remission [54]. These research efforts aim to understand the challenges faced by patients with Wilms tumor compared to those with other pediatric malignancies, focusing in particular on the psychosocial ramifications and late effects of treatment [55].

## 3. Characteristics and Classification of Pediatric Nephroblastoma

Pediatric nephroblastoma (Wilms’ tumor) usually arises from embryonal kidney tissue, displaying several histological features that reflect its developmental origin [56,57]. The tumor is often unilateral, but bilateral cases may occur in about 6% of patients, which is significant for treatment and prognostic considerations [57,58]. Unilateral nephroblastoma accounts for the majority of cases, while bilateral nephroblastoma, although less common, presents unique challenges in diagnosis and treatment. Unilateral nephroblastoma usually manifests as a solitary renal mass, often diagnosed in children between 2 and 5 years of age [59,60]. The standard treatment protocol involves total nephrectomy, which has shown favorable results, especially in localized cases. Studies indicate that event-free survival rates after nephron-sparing surgery (NSS) for unilateral nephroblastoma may be comparable to those following total nephrectomy, suggesting that NSS is a viable option for selected patients [61,62]. However, the prognosis remains dependent on several factors, including tumor histology and stage at diagnosis [63]. In contrast, bilateral nephroblastoma, which occurs in about 4–8% of all Wilms tumors, presents a more complex clinical scenario. This form is often associated with nephroblastomatosis, characterized by the presence of multiple nephrogenic debris, which can complicate surgical management and increase the risk of recurrence [64,65]. Treatment of bilateral nephroblastoma requires a careful balance between achieving oncologic control and preserving renal function, especially given the potential for renal failure after radical nephrectomy [65]. The presence of underlying genetic syndromes, such as Beckwith-Wiedemann syndrome, is also more frequent in bilateral cases, which may influence both tumor biology and therapeutic approach [63,66]. The management of bilateral nephroblastoma often involves preoperative chemotherapy to reduce the tumor burden, followed by a combination of nephrectomy and careful monitoring of residual disease. Research indicates that the biology of bilateral tumors may differ from that of unilateral cases, requiring distinct treatment protocols [65,66]. In addition, long-term survival rates for patients with bilateral nephroblastoma are generally lower than those for unilateral cases, highlighting the need for continued research into more effective treatment strategies [60].

Wilms tumor is characterized by several clinical presentations. The most common symptom is an abdominal mass, which is palpable in about 75–95% of cases at the time of diagnosis [67]. This mass often leads to an increase in abdominal girth, making it a clinically significant marker. In some cases, patients may experience additional symptoms such as hematuria, high blood pressure, or abdominal pain, although these are less common [68,69]. In rare cases, the Wilms tumor may manifest with unusual symptoms such as dilated cardiomyopathy or congestive heart failure, highlighting the tumor’s potential for various presentations [70,71]. The clinical features of Wilms tumor may vary depending on its histologic subtype. For example, botryoid Wilms’ tumor, a rare variant, usually presents as an asymptomatic mass, differentiating it from the more common symptomatic presentations of typical Wilms’ tumor [68,72]. In addition, the presence of venous thrombus is often seen at the same time as the diagnosis, especially in the later stages of the disease [67,73]. This highlights the importance of thorough clinical evaluation and imaging studies in the diagnosis and management of Wilms tumor. In terms of demographic factors, Wilms’ tumor predominantly affects children, with a higher incidence observed in those under 5 years of age [74,75]. Tumor presentation can also be influenced by genetic syndromes, which can lead to bilateral tumors or additional congenital anomalies [76]. The clinical management of Wilms tumor usually involves a combination of surgery and chemotherapy tailored to the stage and histological characteristics of the tumor [77,78]. Treatment sometimes also involves radiotherapy, with an excellent prognosis for localized disease, where survival rates exceed 90% [57,58]. However, the prognosis is significantly poorer for metastatic cases, underlining the importance of early detection and intervention [56,79].

The classification of Wilms tumors can be approached from various angles, including histological subtypes, genetic mutations, and molecular features (Table 1).

In terms of histological classification, Wilms tumors are mainly classified into three histological subtypes: triphasic, biphasic, and monophasic. The triphasic subtype is the most common and comprises three components: blastemal, epithelial, and stromal cells, resembling the architecture of the normal nephron [79,85]. This three-phase pattern is crucial for diagnosis and is a hallmark of the tumor’s embryonic origin [86]. The presence of these components reflects the stage of development and differentiation of the tumor, with variations in their proportions influencing clinical behavior and prognosis [87]. The predominant cell type in Wilms’ tumors is the blastemal cell, which is derived from the metanephric blastema, an essential component in kidney development. The blastemal component is characterized by small, undifferentiated cells with reduced cytoplasm, compared to the epithelial component, which may have primitive tubular structures [88,89]. This type of tumor is the most common kidney tumor in children, accounting for about 80% of pediatric kidney tumors [58]. The blastemal component of Wilms’ tumor is particularly significant as it reflects the embryonic origin of the tumor, consisting of undifferentiated mesenchymal cells, which is often most prominent in nephroblastoma [90]. This component is responsible for the aggressive nature of the tumor as it retains the potential for further differentiation into epithelial and stromal components. Studies have shown that blastemal cells display characteristics of renal developmental markers, indicating their role in tumor pathogenesis [91,92]. The presence of these cells is essential for understanding tumor biology as they are thought to be responsible for tumor growth and differentiation. 

The epithelial component usually has primitive renal tubules, while the stromal component can include a variety of mesenchymal tissues, such as muscle and fat [88]. The interaction between these components is essential for understanding tumor biology and its response to treatment [93]. The stromal component can vary significantly, with some tumors showing a predominance of stromal cells, leading to a biphasic subtype in which blastemal or epithelial components are less prominent. In contrast, monophasic Wilms tumors consist predominantly of a single cell type, usually blastemal cells, and are often associated with a more aggressive clinical course [88,94].

Histologically, nephroblastoma can present with varying degrees of differentiation, which can complicate diagnosis and treatment strategies. For example, tumors may show a predominance of one component over the others, leading to classifications such as ‘blastemal predominant’ or ‘stroma predominant’ nephroblastoma [95]. The presence of teratoid features, in which heterologous tissues such as cartilage or neural tissue are present, may further complicate the histologic landscape of nephroblastoma [96]. Such variations require careful histopathologic evaluation to ensure accurate diagnosis and appropriate management [87]. In addition, the histological classification of Wilms tumor often emphasizes the importance of the blastemal component, as its presence may influence prognosis and treatment strategies [97,98]. In addition to the classical triphasic structure, there are variations of Wilms tumors, including anaplastic Wilms tumors, which are characterized by more aggressive behavior and a higher likelihood of resistance to treatment [99,100]. These tumors may have a predominance of blastemal cells with diffuse anaplasia, which is associated with poor outcomes [100]. Other types of renal tumors that may be confused with Wilms tumor include mesoblastic nephroma and cystic nephroma, which may also occur in pediatric patients but have distinct histologic features and clinical implications [101,102]. 

The classification of Wilms tumors has important clinical implications. The presence of unfavorable histology, such as anaplasia, requires more aggressive treatment strategies, including intensive chemotherapy and possibly nephrectomy [103]. In contrast, tumors classified as favorable histology may respond well to less aggressive treatment regimens [94]. Understanding the histologic and genetic landscape of Wilms tumors can thus guide therapeutic decisions and improve patient outcomes. Wilms’ tumor is classified into five stages, which are essential in determining treatment strategies, as well as in estimating the patient’s prognosis. For example, higher stages have been observed to correlate with unfavorable outcomes, including increased likelihood of metastasis and poor overall survival rates [104,105]. In addition, variable histologic features (anaplasia) may independently affect prognosis and should be considered when selecting treatment modalities [106]. Table 2 presents the staging system of Wilms’ tumor, including the key features of each stage and the associated survival rates.

Although pediatric nephroblastomas are an important topic in the study of pediatric renal tumors, mainly represented by Wilms tumors, there is a spectrum of non-Wilms renal tumors (NWRT), including clear cell renal sarcoma (CCRS), malignant rhabdoid tumors of the kidney (MRTK), congenital mesoblastic nephroma (CMN), renal cell carcinoma (RCC), primary neuroectodermal tumor (PNET) and renal lymphoma [10,112,113]. Non-Wilms tumors of the kidney are a diverse group of pediatric renal neoplasms that are distinct from the more commonly known Wilms tumor. These tumors collectively represent a smaller but clinically important subset of pediatric renal neoplasms, accounting for approximately 7% of all pediatric malignancies [114,115]. Classification of these tumors is essential for diagnosis, treatment, and understanding of their biological behavior. Each of these tumors has unique histologic features, clinical presentations, and treatment protocols. 

Renal cell carcinoma (RCC) is a rare but significant entity in pediatric oncology, often presenting with symptoms such as hematuria and abdominal mass. It is characterized by its origin in the renal tubular epithelium and may present with different histological subtypes, including clear cell and papillary types [113]. Clear-cell renal sarcoma (CCRS) is another notable non-Wilms tumor, usually occurring in children between the ages of 1 and 4 years. It is distinguished by its aggressive nature and specific chromosomal abnormalities, particularly involving chromosome 17 [10,113]. Malignant rhabdoid tumor of the kidney (MRTK) is an extremely aggressive neoplasm that usually affects infants and young children. It is associated with mutations in the SMARCB1 gene and is characterized by its rhabdoid morphology [113]. Congenital mesoblastic nephroma (CMN) is often diagnosed in newborns and infants, presenting as a palpable abdominal mass. In general, it is considered a benign tumor, although it can be aggressive in some cases. Primitive neuroectodermal tumor (PNET) of the kidney is a rare variant that arises from neuroectodermal tissue and can be difficult to differentiate from other small round blue cell tumors [10,113]. Finally, renal lymphoma, although more common in adults, can also occur in children, often presenting as bilateral renal masses [113]. Overall, although Wilms tumor has a relatively lower incidence compared to other pediatric malignancies, understanding Wilms tumor histopathologically is crucial for both pathologists and clinicians, as the majority of renal tumors in children are due to Wilms tumor, making it essential to differentiate it from rare tumors such as clear cell sarcoma of the kidney and malignant rhabdoid tumor [25,52]. 

It has been observed that the development of Wilms’ tumor has been correlated with genetic and environmental risk factors predisposing children to this malignancy. In terms of genetic factors, various mutations have been identified that significantly increase the risk of developing Wilms tumor. Research has identified several genes associated with Wilms’ tumor predisposition, such as *TRIM28*, *FBXW7*, *KDM3B*, and *NYNRIN*. *TRIM28* mutations have been implicated in approximately 8% of familial and 2% of sporadic cases of Wilms tumor [116]. Both the Children’s Oncology Group and other initiatives have elucidated genetic aberrations impacting many pathways crucial for kidney development and oncogenesis, finding that there are recurrent mutations in Wilms tumor of the *WT1*, *CREBBP*, and *MLLT1* genes [94]. Polymorphisms in the *TP53* gene have also been studied, but the results are controversial regarding their specific association with Wilms tumor risk, with some studies suggesting possible links, while others find no semi-significant associations [37,117]. Some studies have explored variants in the LMO1 super-enhancer region LMO1 that may contribute to susceptibility, indicating the complex interplay of genetic factors [36,118]. Although less studied than genetic factors, environmental factors are hypothesized to contribute to Wilms tumor risk. Some studies suggest that maternal hormonal exposures during pregnancy and paternal occupational exposures may have associations with increased Wilms tumor incidence in offspring, although these findings are often not statistically significant [119,120]. In addition, factors such as older parental age, particularly paternal age, have been linked to an increased risk of Wilms tumor through epigenetic alterations that affect gene expression patterns [121,122].

The imaging features of these tumors can vary significantly, complicating the differential diagnosis. For example, while Wilms tumors are often characterized by a large mass with necrotic areas, non-Wilms tumors may present with distinct features such as calcifications or specific vascularization patterns [123]. Understanding these imaging features is essential for accurate diagnosis and management.

## 4. Biomarkers in the Diagnosis of Nephroblastoma

Given the tendency of the disease to metastasize and relapse, the identification of effective biomarkers for the diagnosis and prognosis of nephroblastoma has intensified, also driven by the need to improve outcomes. Disease prognosis is largely based on histopathology and staging at the time of diagnosis. The emergence of biomarkers has introduced a powerful additional approach to improve the prediction of clinical outcomes. Several key biomarkers have been associated with nephroblastoma prognosis, mainly by correlating with cellular and molecular features observed in tumor samples.

Genetically, Wilms tumors show significant heterogeneity. Reported prognostic markers include overexpressed proteins such as Wilms tumor 1 (*WT1*), *Ki67*, and *p53*. Specifically, tumors showing >10% *WT1*, >5% *Ki67*, and >5% *p53* positivity are associated with negative survival outcomes, indicating a more aggressive tumor behavior [106,124]. These findings underline the importance of using immunohistochemical markers to assess tumor behavior and predict survival, highlighting the role of *Ki67* in proliferative assessment, albeit with limited prognostic accuracy in clinical settings [125]. Recent studies have highlighted the role of genetic alterations that play a key role in nephroblastoma pathogenesis, in particular, mutations in key regulatory genes such as *WT1*, *WT2*, *CTNNB1*, *WTX*, and *TP53*, which are associated with the development and progression of nephroblastoma tumors [96,99,126,127]. These genetic alterations (e.g., mutations in *CTNNB1* and *WTX*) or loss of heterozygosity on specific chromosomes (1p, 1q, 11p15, and 16q) have also been linked to nephroblastoma development, highlighting their usefulness in prognostic assessments [128,129]. It has been stated that key mutations (e.g., the inactivation of the *WT1* gene) occur in about 20% of cases, and mutations in *CTNNB1* (β-catenin) are found in about 15% of tumors [99]. *WT1* gene mutations have been implicated in the pathogenesis of Wilms tumor, particularly in syndromic cases such as WAGR syndrome and Denys-Drash syndrome [57,85]. Tumors with *TP53* mutations are often associated with anaplastic histology, which correlates with poor prognosis and chemoresistance [100,130]. These mutations can affect blastemal cell differentiation pathways, further complicating tumor behavior and response to therapy [131,132]. 

In addition to these markers, recent studies have highlighted the prognostic significance of long non-coding RNAs (lncRNAs) and microRNAs in nephroblastoma. The identification of long non-coding RNAs as biomarkers in nephroblastoma suggests a new avenue of research into tumor biology and potential therapeutic targets [133]. For example, *SOX21-AS1* has been identified as an lncRNA that is hypomethylated in neuroblastoma and correlates with the clinical stage, serving as a possible prognostic marker [134,135]. Similarly, the expression and significance of *FOXO3a*, which inhibits nephroblastoma cell proliferation and invasion through down-regulation of the Wnt/β-catenin signaling pathway, presented opportunities for novel therapeutic targets in the management of nephroblastoma [136,137]. In addition, miRNAs, particularly miR-21 and miR-130b, are upregulated in nephroblastoma, suggesting their potential utility as diagnostic markers linked to tumor progression and treatment response [138,139]. Up to 176 miRNAs have been identified as significantly differentially expressed in the serum of nephroblastoma patients, both before and after chemotherapy, marking them as advantageous candidates for diagnostic and prognostic stratification [27].

Recent studies have identified specific molecular biomarkers that can help classify Wilms tumors based on their epigenetic profiles. For example, methylation patterns of genes such as *HIC1* and *BRCA1* can distinguish between predominantly stromal and predominantly epithelial tumors, which are associated with different clinical outcomes [140]. Moreover, *RASSF1* gene methylation status has been shown to correlate with tumor behavior and could serve as a novel biomarker for predicting patient outcomes [141]. In addition, the study of microRNAs, in particular, miR-21 and miR-130b, offers new avenues for understanding tumor biology, as they affect pathways critical for tumor growth and chemoresistance [138]. For example, overexpression of *miR-483-3p* has been linked to promoting invasive features in Wilms tumor cells, highlighting the role of microRNAs as diagnostic biomarkers [138]. 

Proteomic methodologies have also provided insight into serum biomarkers, revealing potentially useful indicators such as haptoglobin and apolipoprotein C-I. Studies indicate that the expression levels of these biomarkers vary according to tumor stage, suggesting their usefulness in predicting treatment responses and prognostic outcomes [142]. Proteins involved in key cellular processes, such as *p53*, *BCL-2*, and *VEGFR1*, have shown prognostic promise due to their role in apoptosis and micro-mediated tumor modulation [26,143]. In addition, the nephroblastoma-related glycoprotein glypican-3 (GPC3) may serve as a diagnostic marker, especially when its expression in certain tumor types is considered [144]. Identifying and quantifying these biomarkers in the tumor microenvironment or by liquid biopsy provides new methodologies for monitoring disease progression and treatment response [141,145]. 

The role of the tumor microenvironment also contributes to biomarker discovery. Myelin and lymphocyte myelin (MAL) protein has been suggested as a prognostic biomarker due to its implications in the interaction between the tumor and its microenvironment [26]. In addition, the development of competitive endogenous RNA (ceRNA) networks has provided insights into the molecular interactions underlying nephroblastoma progression, highlighting the complexity of tumor biology and the potential for integrating multi-omics data to refine diagnostic and prognostic models [146].

Immunologic profiling of nephroblastoma complicates the biomarker landscape. Research has identified autoantibody signatures that can differentiate nephroblastoma from similar malignant tumors, such as neuroblastoma, with high sensitivity and specificity, requiring further investigation of autoantibody profiles for reliable diagnostic tools [147]. 

Overall, understanding nephroblastoma and its various cellular components is essential for the development of targeted therapies and improved prognostic models [148]. The diagnosis and prognosis of nephroblastoma are increasingly linked to efforts to discover and validate biomarkers. The dynamic interplay between histopathologic features, genetic and proteomic alterations, miRNA profiles, and immune signatures presents a richly layered approach that may transform clinical management and therapeutic outcomes in this complex pediatric malignancy.

### Early Detection Screening of Wilms’ Tumor 

Screening for the early detection of Wilms tumor, the most common malignant tumor of the kidney in children, is particularly important for children at increased risk due to genetic predisposition, associated syndromes or family history. Surveillance strategies largely advocate abdominal ultrasound as the preferred initial imaging method due to its non-invasive nature and sensitivity in detecting renal abnormalities. Khanna et al. state that although CT scans are also used in the evaluation of Wilms tumor, ultrasound is favored in a screening context for its ability to monitor vascular structures, making it the optimal tool for screening high-risk children [149]. 

Current practice suggests that children with conditions that significantly increase the risk of Wilms’ tumor, such as syndromic abnormalities, should undergo regular imaging examinations. Specifically, for these high-risk populations, ultrasound screening every three months, at least up to the age of 8 years, has been recommended to facilitate early detection, thereby improving prognosis and allowing more tailored treatment strategies [150]. Children with predisposing syndromes such as Beckwith-Wiedemann syndrome (BWS), Denys-Drash syndrome (DDS) and horseshoe-shaped renal anomalies have a significantly higher incidence of Wilms tumors. Therefore, surveillance protocols for these high-risk groups are not only advisable but necessary. The study by Bach et al. indicates that although survival rates of children with BWS and Wilms tumor are not significantly different from those without BWS, those with BWS who develop Wilms tumor are usually diagnosed at earlier stages, which may reduce treatment-related morbidity [151]. Furthermore, Gariépy-Assal et al. emphasize the need for regular monitoring of Wilms tumor in children with DDS. This monitoring is essential because recurrence rates for Wilms tumor vary according to its histologic type, necessitating close surveillance [152]. Perlman syndrome, although less common, also requires specific screening guidelines. According to a study by Demırel et al., a significant proportion (32%) of children with Perlman syndrome develop Wilms tumors, highlighting the need for abdominal ultrasound every three months until the age of five [153]. 

Genetic testing is also a fundamental component of screening for children who are more likely to develop Wilms tumors. In particular, germline testing is primarily recommended for those who pre-screen for high-risk histologic features, bilateral tumors, or syndromic features. Research indicates that this genetic component should also extend to children with predominantly epithelial histology, a subtype that is less common but still requires vigilant monitoring due to its association with genetic predisposition [154]. The role of genetic factors strengthens the case for screening for this pathology in co-pupils, with specific genetic mutations being associated with an increased risk of Wilms tumor. The study by Fu et al. identifies polymorphisms in *KRAS* and *NRAS* genes as linked to Wilms tumor susceptibility, suggesting potential targeted screening strategies for those with identified mutations [155].

Given that pediatric Wilms tumor accounts for approximately 90% of all renal tumors diagnosed in children and with a notable increase in survival rates in recent decades, proactive measures for high-risk populations are imperative. Collaborations between pediatric oncologists, radiologists, geneticists, and children’s families are essential in forming a coherent screening protocol that addresses both physical manifestations and family concerns. 

## 5. Current Treatment Therapies for Wilms’ Tumor

Wilms’ tumor or nephroblastoma is a pediatric renal malignancy that requires a multifaceted therapeutic approach, primarily involving surgery, chemotherapy, and, in some cases, radiotherapy [156,157]. 

The cornerstone of treatment is surgical intervention. Most often, radical nephrectomy (removal of the affected kidney) is performed, along with the resection of regional lymph nodes. In cases of bilateral Wilms tumor, partial nephrectomy may be considered to preserve renal function when feasible. Following surgery, adjuvant chemotherapy is typically administered. Intensive multiagent chemotherapy regimens are used for more aggressive tumors and advanced diseases. For localized cases, chemotherapy helps eliminate any remaining tumor cells post-surgery. Cure rates for low-stage disease are notably high, ranging from 85 to 95 percent. Radiation therapy is usually reserved for children with more advanced disease (Stage III) or when there are distant metastases, such as in the lungs. It may be combined with chemotherapy to improve overall outcomes. There is an increasing interest in newer therapeutic strategies, including immunotherapy and minimally invasive surgical techniques. These options aim to reduce the side effects associated with traditional treatments and improve the quality of life for pediatric patients. Recent studies have begun exploring biomarkers and biological therapies to assess their potential as less risky alternatives compared to conventional treatments [129,158,159].

Treatment protocols for Wilms tumor have evolved significantly over the years, with the National Wilms Tumor Study Group (NWTSG) protocol, the International Society of Pediatric Oncology (SIOP) protocol, and the recommendations of the Polish Pediatric Solid Tumor Treatment Group (PPGGL) being the prominent management strategies. The NWTSG typically advocates initial surgery followed by adjuvant chemotherapy. At the same time, the SIOP recommends neoadjuvant chemotherapy to reduce tumor size before surgical resection, particularly in patients older than six months [156,157]. Table 3 presents a comparison of the current treatment protocols according to these organizations.

The NWTSG protocol emphasizes radical nephrectomy as the cornerstone of treatment, allowing for accurate tumor staging and histologic evaluation. After surgery, patients receive adjuvant chemotherapy, which often includes agents such as actinomycin D and vincristine. This regimen has been shown to significantly improve survival rates, with five-year survival rates for localized Wilms tumors exceeding 90% [161]. In contrast, the neoadjuvant approach of the SIOP protocol aims to shrink the tumor before surgery, thereby minimizing the risk of intraoperative complications such as tumor spread. This strategy has been particularly beneficial for patients with large tumors or those with vascular involvement, as it facilitates a more complete surgical resection [162].

Recent studies have highlighted the role of immunotherapy and targeted therapies in managing Wilms tumor. For example, Zhao et al. reported a case of metastatic Wilms tumor in patients treated with a combination of chemotherapy, immunotherapy, and targeted therapy, suggesting that an immune-engaged tumor microenvironment may enhance treatment efficacy [163]. This pilot study indicated that blockade of the PD-1/PD-L1 pathway could improve outcomes in patients with Wilms tumor, particularly those with advanced disease, highlighting the potential of immunotherapeutic strategies in this setting.

In addition to traditional chemotherapy, there is a growing interest in using new agents and combination therapies. For example, Venkatramani et al. explored the efficacy of a regimen combining vincristine, irinotecan, temozolomide, and bevacizumab in patients with multiple relapsed Wilms tumors. This study demonstrated that combination therapy could reduce tumor size and attenuate the risk of metastases, offering a promising pathway for patients with refractory disease [164]. The study findings suggest that antiangiogenic therapy, when used in combination with standard chemotherapeutic agents, may improve treatment outcomes in relapsed cases.

The role of radiation therapy in the treatment of Wilms tumor remains a topic of ongoing research and debate. Although the COG and SIOP protocols include radiotherapy for high-risk and relapsed cases, their availability is limited in many low-resource settings, as emphasized by Holmes et al. in their study of treatment outcomes in Malawi [157]. The authors noted that despite advances in chemotherapy protocols, radiotherapy dependence persists for certain patient populations, emphasizing the need for equitable access to comprehensive cancer care. In addition, the identification of molecular markers and genetic predisposition has opened new avenues for targeted therapies in Wilms tumor [138,165]. These findings highlight the importance of integrating molecular biology into the treatment paradigm for Wilms tumor.

Wilms tumor management also requires careful consideration of the long-term effects of treatment. Complications following radical nephrectomy and subsequent therapies can have a significant impact on the quality of life of survivors. For example, Sethasathien et al. reported a case in which dilated cardiomyopathy developed in a post-nephrectomy patient, emphasizing the need for continuous monitoring and supportive care in this population [70]. As treatment protocols continue to evolve, increasing emphasis is being placed on minimizing treatment-related morbidity while maximizing therapeutic efficacy.

**Figure 1:**
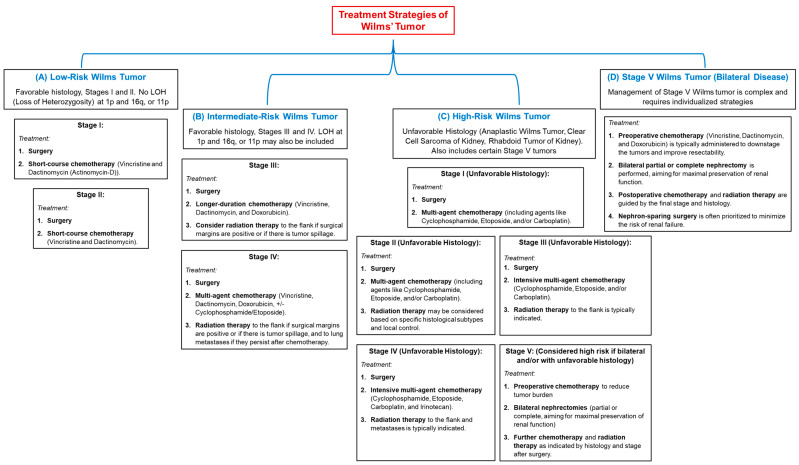
Treatment Protocols by Risk Group and Stage of Wilms Tumor.

Overall, the current treatment landscape for Wilms tumor is characterized by a multimodal approach integrating surgery, chemotherapy, and emerging therapies such as immunotherapy and targeted agents. The choice of treatment protocol (NWTSG, SIOP, or PPGGL) depends on various factors, including tumor stage, patient age, and specific clinical circumstances (Figure 1).

Survival outcomes for Wilms tumor are significantly influenced by several clinical factors, including tumor stage, histology and presence of metastases at diagnosis [166,167,168]. For example, children diagnosed with Diffuse Anaplastic Wilms’ Tumor (DAWT) have particularly poor survival outcomes, as evidenced by a 4-year recurrence-free survival rate that can drop to around 40% [167]. The application of Kaplan-Meier survival analysis was essential for understanding these survival trends. Historically, the 5-year survival rate for Wilms tumor has been reported to be over 90% for patients with favorable histology. Specifically, studies indicate that for children with stage I Wilms tumors with favorable histology, the reported 5-year survival rate is approximately 97.6% [152]. This reflects evolving treatment strategies that have shifted to multi-modal approaches involving surgery followed by appropriate adjuvant chemotherapy. In North America, the general consensus in the treatment protocol is to initiate treatment with surgical resection prior to any chemotherapy, aligning with the guidelines of the National Wilms Tumor Study Group (NWTSG) [169,170]. In contrast, the survival outcomes of children diagnosed with advanced Wilms disease or with unfavorable histology reflect a worrying disparity. For example, survival estimates for children with stage III and IV tumors are significantly lower, at around 75% and 65% respectively [171]. In addition, children with anaplastic features or those with relapsed disease often experience significant resistance to treatment, leading to very low survival rates, which underlines the heterogeneity of this disease [172,173]. The disparity in survival rates is also notable when comparing different age groups; for example, children older than 10 years have a considerably poorer prognosis, with overall survival rates reported at only 63% [101]. 

The impact of socio-economic factors and geographical location on survival rates is also highlighted. Studies of different populations show that survival statistics are very different between high- and low-income countries. For example, survival rates for pediatric Wilms tumors in Malawi have been reported to be as low as 46% due to factors such as treatment dropout and high treatment-related mortality rates [46,174]. Reports from sub-Saharan Africa highlight that 5-year survival rates fluctuate dramatically depending on local treatment capacities, dropping as low as 11% in Sudan, mainly due to failures to complete treatment [175]. By contrast, children treated in Western countries, where treatment protocols and access to care are more robust, have survival rates of over 90% [171,176,177]. In addition, the introduction of comprehensive treatment protocols has significantly influenced survival rates, with numerous clinical trials leading to refined therapeutic strategies incorporating surgery, chemotherapy, and radiotherapy where indicated [168,177]. The impact of comprehensive therapeutic approaches is illustrated by studies specifically analyzing data from multinational collaborations, demonstrating how advances in treatment protocols have led to improved survival in different demographic settings [47,177].

## 6. Alternative Treatment Therapies

Recent studies have examined the role of modifying existing chemotherapy regimens to improve outcomes in Wilms tumor. For example, Chen et al. explored the role of *UNC13B* in modulating the sensitivity of Wilms tumor cells to doxorubicin, a common chemotherapeutic agent. This study used an in vitro model to evaluate the effects of *UNC13B* on lysosomal function and drug resistance in Wilms tumor cell lines. Findings indicated that the down-regulation of *UNC13B* increased the sensitivity of these cells to doxorubicin, suggesting a potential target for overcoming chemoresistance [178].

In addition to conventional chemotherapy, targeted therapies are being explored as alternative treatments for Wilms’ tumor. For example, Zhang et al. investigated the role of *circ-PRMT5* in promoting the proliferation of Wilms’ tumor cells through the *miR-7-5p/KLF4* axis. This study utilized both in vitro and in vivo models, demonstrating that inhibition of *circ-PRMT5* significantly reduced tumor growth and enhanced the sensitivity of Wilms’ tumor cells to chemotherapy. These findings suggest that *circ-PRMT5* may serve as a promising therapeutic target for improving treatment responses in patients with Wilms’ tumor [179]. In addition, expression of liver regenerating phosphatase-3 (PRL-3) has been linked to poor prognosis in Wilms tumor. Sun et al. conducted a cohort study that analyzed *PRL-3* expression levels in tumor samples from patients. The results indicated that elevated *PRL-3* expression was associated with increased recurrence rates, suggesting that *PRL-3* could be a potential biomarker for identifying high-risk patients who may benefit from more aggressive treatment strategies [180].

Molecularly targeted therapies represent a promising avenue for treating Wilms tumor, especially in cases with unfavorable histological features or recurrent disease. A study by Li et al. investigated the role of *microRNA-215-5p* in Wilms tumor, demonstrating its ability to inhibit cell proliferation and migration by targeting *CRK* proto-oncogene. This in vitro study used human Wilms tumor cell lines and showed that overexpression of *miR-215-5p* significantly reduced tumor cell viability, suggesting its potential as a therapeutic target. The findings highlight the importance of understanding the molecular basis of Wilms tumor to develop effective targeted therapies [181]. Another study by Yu and Liu focused on the long non-coding RNA LINC01339, which was found to prevent Wilms tumor development through the *miR-135b-3p/ADH1C* axis. This research utilized both in vitro and in vivo models, indicating that modulating this pathway could offer a novel therapeutic strategy. The study results suggest that targeting specific RNA molecules may offer a complementary approach to conventional therapies, potentially improving outcomes for Wilms tumor patients [182].

Immunotherapy is gaining space as an alternative treatment for various malignancies, including Wilms tumor. The expression of neural cell adhesion molecules (NCAMs) in Wilms’ tumors has been associated with poor prognosis, as Kapur and Rakheja pointed out. Their study suggests that NCAM could serve as a therapeutic target, particularly in immunotherapeutic strategies aimed at enhancing antitumor immunity [183]. The potential of therapies targeting NCAM is further supported by evidence from other cancers, indicating that similar approaches could be beneficial in Wilms tumor. A case report by Lee et al. described the successful treatment of a stage IV Wilms tumor using a combination of herbal Korean medicine, hyperthermia therapy (oncothermia), and thymosin-α1 treatment (Zadaxin i.m.) [184]. This integrative approach aimed to enhance the immune response while reducing side effects associated with conventional treatments. The patient showed a positive response, suggesting that combining traditional therapies with modern immunotherapeutic strategies can improve patient outcomes and reduce the burden of treatment.

Chemoresistance remains a significant challenge in Wilms tumor management, especially in recurrent cases. Research by Che et al. identified *microRNA-483-3p* as a key regulator of chemoresistance in Wilms tumor cells, promoting proliferation, migration, and invasion. This study used in vitro models to demonstrate that targeting *miR-483-3p* could enhance the sensitivity of Wilms tumor cells to chemotherapeutic agents such as doxorubicin. The findings highlight the potential to develop novel agents that can overcome resistance mechanisms, thereby improving treatment efficacy [138]. In addition, Venkatramani et al. reported the use of a combination chemotherapy regimen involving vincristine, irinotecan, temozolomide, and bevacizumab for multiple relapsed Wilms tumors. This case report illustrated the potential of using novel drug combinations to manage relapsed disease. The study highlights the need for continued research into alternative chemotherapeutic strategies that can address the complexities of Wilms tumor treatment [164].

## 7. Future Research Directions

One promising area of investigation is the exploration of genetic and epigenetic alterations associated with Wilms tumor. Recent studies have identified key mutations in various genes, which are implicated in the tumorigenesis of Wilms tumor [94,128]. Furthermore, the role of promoter methylation has emerged as a potential biomarker for predicting patient outcomes [185]. Future research should focus on large-scale genomic studies to elucidate the full spectrum of genetic alterations in Wilms tumor, which could lead to the identification of novel therapeutic targets and prognostic markers.

Another significant avenue for future research is the investigation of the tumor microenvironment and its influence on Wilms tumor progression and treatment response. The expression of microRNAs has been linked to chemoresistance in Wilms tumor cells, indicating that the tumor’s biological behavior may be modulated by its microenvironment [138]. Understanding the interactions between tumor cells and their microenvironment could inform the development of combination therapies that enhance treatment efficacy.

Moreover, the exploration of immunotherapy as a treatment modality for Wilms tumor is gaining traction. Recent studies have shown promise in utilizing adoptive T-cell therapies targeting tumor-associated antigens [186]. Future clinical trials should aim to evaluate the safety and efficacy of these immunotherapeutic strategies in pediatric populations, potentially offering new hope for patients with relapsed or refractory disease.

Finally, the investigation of rare variants of Wilms tumor, such as teratoid Wilms tumor, warrants further attention. These atypical presentations may have distinct biological behaviors and treatment responses, highlighting the need for dedicated research to understand their unique characteristics and optimize management strategies [187].

## 8. Conclusions

Nephroblastoma (Wilms’ tumor) is a complex pediatric renal tumor with significant implications for the child’s health and a notable incidence of unilateral and bilateral forms. While unilateral nephroblastoma is more common and generally has a better prognosis, bilateral nephroblastoma presents significant challenges due to its complex biology and treatment requirements. This complex malignancy with distinct features requires a thorough understanding of its biological behavior, genetic associations, and the application of effective treatment protocols to optimize patient outcomes. The classification of pediatric nephroblastomas and their non-Wilms counterparts is multifaceted, involving histologic, genetic, and imaging considerations. Integration of histological and molecular data is essential to optimize treatment strategies and appropriate prognostic assessments in affected children. Based on histological features, Wilms’ tumors can be classified into triphasic, biphasic, and monophasic subtypes, while genetic mutations such as those in *WT1*, *CTNNB1*, and *TP53* refine this classification. The triphasic histologic structure comprises blastemal, epithelial, and stromal components, with blastemal cells being the most significant in terms of tumor biology and prognosis. Understanding the interplay between these components, together with the genetic factors involved, is essential for the effective diagnosis and treatment of this pediatric malignancy. Understanding these cell types and their differentiation from other renal tumors is essential for effective diagnosis and treatment strategies in pediatric oncology. The screening process for Wilms’ tumors in high-risk children involves a combination of regular imaging protocols and genetic testing, particularly targeting those with identifiable risk factors or associated syndromes. This methodical approach aims to increase early detection and improve overall treatment outcomes for this pediatric cancer. Multidisciplinary approaches involving genetic counseling can help families understand the risks associated with hereditary cancer syndromes by informing them about the necessary surveillance. Surveillance programs, in particular ultrasound screening, together with genetics-based strategies, create a solid framework for early diagnosis and intervention, thereby improving outcomes and reducing the burden of treatment complications. The clinical implementation of biomarkers is under continuous development, with extensive ongoing research aimed at establishing a standardized set of markers for clinical use. The improvement in survival rates for nephroblastoma has been paralleled by progress in biomarker-based therapies and treatments. However, significant disparities remain, particularly in patients with high-risk histology such as diffuse anaplastic Wilms tumor, where survival rates from recurrence remain low. Finally, the current literature emphasizes the need to develop integrative models incorporating clinical, pathological, and molecular data to ensure holistic patient management plans. Emerging techniques in digital analytics and computational biology may enhance the ability of clinicians to discern relevant patterns and associations within biomarker profiles, thus enabling more efficient risk stratification and therapeutic decision-making. The incorporation of biomarkers into clinical protocols represents not only a breakthrough in the management of nephroblastoma but also signifies a broader paradigm shift towards personalized oncology in pediatric cancer care. 

## Figures and Tables

**Table 1:** Classification of Wilms’ tumor according to histological subtypes, genetic mutations, and molecular features [48,80,81,82,83,84].

Classification Category	Subtype/Feature	Description	Clinical Significance/ Prognostic Value
Histological Subtypes	Favorable Histology (FH)	Characterized by well-differentiated blastemal, epithelial, and stromal components without anaplasia.	Generally associated with a good prognosis.
	Anaplastic Histology (AH)	Presence of large, hyperchromatic nuclei with multipolar mitoses. It can be focal (localized) or diffuse (widespread).	Associated with a less favorable prognosis, especially in diffuse anaplasia. Requires more aggressive treatment.
	Clear Cell Sarcoma of Kidney (CCSK)	A distinct histological subtype characterized by cells with clear cytoplasm and a prominent vascular network.	Generally more aggressive than an FH Wilms tumor. Requires specific treatment protocols, including anthracycline-based chemotherapy.
	Rhabdoid Tumor of Kidney (RTK)	Highly aggressive tumors are composed of cells with eccentric nuclei, prominent nucleoli, and eosinophilic cytoplasm, and they often display a loss of *INI1*/*SMARCB1* expression.	Very poor prognosis. Often presents at advanced stages and is associated with central nervous system involvement. Requires intensive multi-modal therapy.
	Congenital Mesoblastic Nephroma (CMN)	Typically diagnosed in infants < 1 year. Usually presents as a large, solid renal mass. Characterized by spindle cells.	Usually favorable prognosis, especially when completely resected. Cellular variants may be more aggressive.
	Wilms Tumor with Regressive Features	Tumors showing regressive changes like necrosis, hyalinization in the stroma, and/or cystic changes.	Associated with good prognosis.
Genetic Mutations	*WT1* mutations	Mutations in the *WT1* gene (Wilms Tumor 1) on chromosome 11p13.	Associated with certain syndromes (e.g., WAGR, Denys-Drash, Frasier) and increased risk of Wilms tumor. It can influence treatment response.
	*CTNNB1* mutations	Mutations in the *CTNNB1* gene (beta-catenin) are found in a subset of Wilms tumors, particularly those with epithelial predominance.	It may be associated with specific histological features and potentially influence prognosis.
	*TP53* mutations	Mutations in the *TP53* gene (tumor protein p53) are often associated with anaplastic histology.	Generally associated with a poorer prognosis and increased risk of relapse.
	*MYCN* amplification	Amplification of the *MYCN* oncogene.	Associated with aggressive tumor behavior and poorer outcomes. More commonly seen in relapsed Wilms tumors.
	*SIX2* and *SIX1*	Overexpression of *SIX2* and *SIX1* genes.	Involved in early kidney development. Overexpression leads to increased blastemal proliferation.
	Loss of heterozygosity (LOH) at 1p and 16q	Loss of genetic material on chromosomes 1p and 16q.	Associated with increased risk of relapse or adverse outcomes.
Molecular Features	Loss of Imprinting (LOI) at 11p15	Loss of imprinting at the 11p15 region affects genes like *IGF2* and *H19*.	Common in Wilms tumors and can contribute to tumor development.
	MicroRNA (miRNA) expression profiles	Specific miRNA expression patterns can differentiate between FH and AH tumors and predict prognosis.	Potential biomarkers for risk stratification and targeted therapy.
	Aberrant Wnt signaling	Activation of the Wnt signaling pathway, often through *CTNNB1* mutations.	Drives cell proliferation and survival. Potential target for therapeutic intervention.

**Table 2:** Wilms’ tumor staging [104,106,107,108,109,110,111].

Stage	Description	Key Features	Approximate 5-Year Overall Survival
I	The tumor is limited to the kidney and completely resected. The tumor is confined within the renal capsule and has not spread to surrounding tissues or lymph nodes. The renal capsule is intact, and there is no rupture of the tumor before or during surgery.	Complete surgical resection; tumor confined to the kidney; no involvement of renal sinus vessels; no spillage of tumor during surgery.	>95%
II	The tumor extends beyond the kidney but is completely resected. The tumor has grown beyond the kidney but has been completely removed surgically. This may include penetration through the renal capsule into the perirenal tissues or involvement of the renal sinus blood vessels. There is no residual tumor left behind after surgery.	The tumor extends beyond the kidney but is completely resected; penetration of the renal capsule involvement of the renal sinus vessels; no residual tumor after surgery. Lymph nodes may or may not be involved, but if they are, the surgeon must completely remove them.	>90%
III	The tumor extends beyond the kidney and is not completely resected. The tumor has spread beyond the kidney and cannot be completely removed surgically. This may be due to tumor spillage during surgery, the involvement of regional lymph nodes that cannot be completely resected, or tumor invasion into adjacent organs or structures.	Incomplete surgical resection; tumor spillage during surgery; involvement of regional lymph nodes that cannot be completely resected; tumor invasion into adjacent organs (e.g., bowel, muscles).	80–90%
IV	Distant metastases are present. The tumor has spread to distant organs, such as the lungs, liver, bone, or brain.	Distant metastases to the lungs, liver, bone, brain, or other organs.	70–80%
V	Bilateral Wilms’ tumor: the tumor is present in both kidneys at the time of diagnosis. Staging is assigned to each kidney individually after biopsy and/or resection.	Tumors are present in both kidneys. Initial therapy often involves chemotherapy to shrink the tumors, followed by bilateral partial nephrectomies to preserve renal function.	>70% (dependent on individual staging of each kidney)

**Table 3:** Comparison of the current treatment protocols for Wilms tumor according to NWTSG, SIOP, and PPGGL [156,157,160].

	NWTSG	SIOP	PPGGL
1. Initial Treatment Approach	Recommends initial surgical resection of the primary tumor before chemotherapy. This approach is aimed at effective local control and reducing the tumor burden before systemic treatment.	Advocates for administering chemotherapy for 4 weeks before surgery, called preoperative chemotherapy. This strategy is intended to shrink the tumor and reduce the risk of tumor rupture during surgery.	Aligns more closely with SIOP protocols, generally favoring preoperative chemotherapy for similar reasons, while adapting treatment based on individual patient circumstances and outcomes.
2. Chemotherapy Regimens	Employs multi-agent chemotherapy consisting of drugs like Actinomycin D (ACT), Vincristine (VCR), and Doxorubicin (ADM). The duration and intensity of treatment depend on the stage of the disease, typically involving a longer regimen for higher-stage tumors.	Utilizes a similar array of chemotherapeutics as NWTSG but allows for some variation in treatment duration and dosing. For lower-stage tumors, less aggressive combinations may be used, whereas higher stages receive more intensive regimens. The SIOP protocols also dictate the timing of postoperative chemotherapy based on initial staging and response.	Treatment regimens are tailored to patient needs, often referencing SIOP guidelines. The chemotherapeutic agents and their dosages are adapted according to tumor histology and response to initial treatment, keeping in mind the higher toxicity levels seen in adult patients compared to children.
3. Radiotherapy Recommendations	Radiotherapy is primarily recommended for advanced stages (III and IV) or for cases with unfavorable histology. Standard doses range from 10 to 20 grays (Gy) depending on the tumor stage and characteristics.	Similar to NWTSG, SIOP recommends radiotherapy mainly for advanced cases and some earlier stages, depending on the tumor’s histological features. The dosage is adjustable, with frequent doses seen between 10 and 15 Gy or more for specific cases.	Follows principles similar to SIOP but may have specific adaptations based on the outcomes of the Polish cohort. The use of radiotherapy is selectively based on individual patient circumstances, often after the assessment of chemotherapy responses.
4. Prognostic Factors and Follow-Up	Uses a detailed classification system based on histological features for risk stratification, impacting treatment decisions and follow-up protocols.	Additionally, it emphasizes prognostic factors like response to preoperative chemotherapy, influencing not just treatment plans but also ongoing monitoring and potential adjustments.	Establishes follow-up protocols that consider both clinical and biological prognostic factors, focusing on long-term outcomes and quality of life for adult patients transitioning from pediatric protocols.

## Data Availability

Not applicable.

## Abbreviations

The following abbreviations are used in this manuscript:

BWS	Beckwith-Wiedemann syndrome
CCRS	Clear-cell renal sarcoma
CMN	Congenital mesoblastic nephroma
CT	Computer tomograph
DAWT	Diffuse Anaplastic Wilms’ Tumor
DDS	Denys-Drash syndrome
ISPO/SIOP	International Society of Pediatric Oncology
lncRNAs	long non-coding RNAs
MAL	Myelin and lymphocyte myelin protein
MRTK	Malignant rhabdoid tumor of the kidney
NCAMs	Neural cell adhesion molecules
NSS	Nephron-sparing surgery
NWRT	Non-Wilms renal tumors
NWTS	National Wilms Tumor Study
PNET	Primitive neuroectodermal tumor
PPGGL	Polish Pediatric Solid Tumor Treatment Group
RCC	Renal cell carcinoma
